# Probenecid-Mediated Pannexin-1 Inhibition Preserves βFGF-Driven Regenerative Responses in Human Dermal Fibroblasts

**DOI:** 10.3390/ijms27125155

**Published:** 2026-06-06

**Authors:** Ricardo Ceriani, Jaime Maripillan, Stefany Ordenes, Carolina Flores-Muñoz, Joel Novoa-Molina, Andrea Tapia-Bustos, Caroline Weinstein-Oppenheimer, Agustín D. Martínez

**Affiliations:** 1 Laboratorio de Innovación Terapéutica y Diagnóstico Molecular, Escuela de Química y Farmacia, Facultad de Farmacia, Universidad de Valparaíso, Valparaíso 2340000, Chile; jaime.maripillan@uv.cl (J.M.); caroline.weinstein@uv.cl (C.W.-O.); 2 Centro para la Investigación Traslacional en Neurofarmacología (CITNE), Universidad de Valparaíso, Valparaíso 2340000, Chile; andrea.tapia@uv.cl; 3 Laboratorio de Comunicación Intercelular, Instituto de Neurociencia, Facultad de Ciencias, Universidad de Valparaíso, Valparaíso 2340000, Chile; stefany.ordenes@gmail.com (S.O.); cfloreu@uc.cl (C.F.-M.); joel.novoa@postgrado.uv.cl (J.N.-M.); 4 Centro Interdisciplinario de Neurociencia de Valparaíso, Instituto de Neurociencia, Facultad de Ciencias, Universidad de Valparaíso, Valparaíso 2340000, Chile; 5 Synaptopathies Laboratory, Facultad de Medicina, Universidad de Valparaíso, Valparaíso 2340000, Chile; 6 Programa de Doctorado en Ciencias, Mención Neurociencia, Universidad de Valparaíso, Valparaíso 2340000, Chile; 7 Facultad de Ciencias Fisiológicas, Pontificia Universidad Católica de Santiago de Chile, Santiago 8320000, Chile; 8 Programa de Doctorado en Ciencias, Mención Biofísica y Biología Computacional, Universidad de Valparaíso, Valparaíso 2340000, Chile; 9 Centro de Investigación, Desarrollo e Innovación en Productos Bioactivos (Cinbio), Universidad de Valparaíso, Valparaíso 2340000, Chile

**Keywords:** pannexin-1, probenecid, βFGF, dermal fibroblasts, chronic wounds, extracellular ATP, calcium signaling, wound healing

## Abstract

Chronic wounds are characterized by persistent inflammation and impaired fibroblast function, leading to defective tissue repair. Pannexin-1 (Panx1) channels regulate extracellular ATP release and purinergic signaling, processes implicated in inflammation and regeneration. Probenecid (PBN), a clinically approved Panx1 channels inhibitor, has emerged as a potential therapeutic modulator in chronic wounds; however, its compatibility with growth factor-mediated regenerative responses remains unclear. Here, we evaluated whether pharmacological inhibition of Panx1 channels alters βFGF-induced responses in human neonatal dermal fibroblasts (HDFn). Cells were treated with βFGF (10 ng/mL) in the presence or absence of PBN (200 μM), and migration, proliferation, extracellular matrix-related gene expression, extracellular ATP release, intracellular Ca^2+^ signaling, and Panx1 expression/localization were assessed. PBN did not significantly alter βFGF-induced migration, proliferation, or most extracellular matrix-related transcriptional responses, except for a further reduction in *COL1A1* expression. In addition, βFGF did not modify *PANX1* transcript levels, Panx1 protein abundance, or membrane localization. Although βFGF reduced extracellular ATP release and transiently modulated ATP-induced Ca^2+^ signaling, these effects occurred without detectable changes in Panx1 expression or localization. These findings support the compatibility of PBN with βFGF-driven regenerative responses in HDFn cells.

## 1. Introduction

The skin is the most exposed organ to environmental stressors that threaten its integrity [1]. Nevertheless, it possesses a remarkable capacity for self-repair, activating mechanisms to restore lost cellular structures and tissue layers upon injury. Wound healing is a tightly regulated process that involves the coordinated action of inflammatory cells, chemokines, cytokines, extracellular matrix (ECM) molecules, and growth factors [2]. However, conditions such as diabetes, obesity, and aging predispose individuals to chronic wounds—skin ulcers that fail to complete the reparative process [3]. These wounds are typically stalled in the inflammatory phase, leading to an imbalance between pro- and anti-inflammatory signals that impairs the proliferation and migration of resident cells [4]. Chronic wounds are a significant clinical burden, affecting an estimated 1–2% of the population in developed countries, a prevalence that increases to nearly 5% in the elderly [5], whose proportion worldwide is expected to more than double by 2050 [6].

Dermal fibroblasts play a central role in wound healing, particularly during the transition from the inflammatory to the proliferative phase. They migrate into the wound bed from the surrounding dermis, degrade the fibrin clot through matrix metalloproteinases (MMPs), and replace it with ECM proteins such as collagen (Col) and fibronectin (FN) [7]. Subsequently, fibroblasts differentiate into myofibroblasts, which contribute to wound contraction and promote angiogenesis by releasing Vascular Endothelial Growth Factor (VEGF) and Platelet-Derived Growth Factor (PDGF) [8,9]. In chronic wounds, however, fibroblast function is severely impaired: their proliferation, migration, and ECM production are dysregulated, and many adopt a senescent phenotype that exacerbates tissue damage [10].

Emerging evidence suggests that Panx1 channels play a pivotal role in this pathological context. By mediating ATP release, Panx1 channels acts as a central regulator of inflammatory signaling, activating purinergic receptors and promoting inflammasome-dependent cytokine production [11,12,13]. Panx1 channels have been implicated in multiple inflammatory conditions, including infection, vascular injury, and systemic inflammation [11,14,15,16], thereby establishing them as promising therapeutic targets in chronic wounds. In the skin, Panx1 channels are highly expressed in keratinocytes and fibroblasts [17,18,19], where they regulate cutaneous homeostasis and cell behavior. Notably, Panx1 channels inhibition or genetic deletion enhances keratinocyte and fibroblast migration, alters fibroblast differentiation into myofibroblasts, regulates keratinocyte differentiation, and modulates cytoskeletal reorganization [19,20,21], underscoring its role as a gatekeeper of wound repair dynamics.

Probenecid (PBN), a long-standing drug approved for the treatment of gout and hyperuricemia [22], has recently gained attention as a pharmacological inhibitor of Panx1 channels [23]. Through this mechanism, PBN has shown protective effects in diverse pathological models, including bacterial pneumonia [24], demyelination in multiple sclerosis [25], and pulmonary ischemia–reperfusion injury, as well as modulation of inflammatory responses in cardiovascular diseases [24,26]. In the skin, PBN reduces the tumorigenic potential of melanoma cells [27] and promotes dermal fibroblast migration [21], suggesting its potential as a therapeutic agent for wound healing by inhibiting Panx1 channels. However, despite increasing interest in Panx1 channels signaling during tissue repair, the effects of pharmacological inhibition of Panx1 channels by probenecid on the functional responses of human dermal fibroblasts have not been characterized previously.

On the other hand, βFGF is a well-known regulator of fibroblast biology and wound healing [28] as well as antifibrotic processes [29]. In line with its therapeutic relevance, exogenous βFGF has been shown to accelerate cutaneous wound repair *in vivo* robustly [30], which promotes proliferation, survival, migration, angiogenesis, and stimulates VEGF production [31,32,33], and modulates ECM protein synthesis, including FN and collagens I and III (Col I and III) [34,35]. Despite these established roles, the potential interplay between Panx1 channels signaling and βFGF-driven fibroblast responses remains unexplored.

Therefore, this study aimed to evaluate whether pharmacological inhibition of Panx1 channels with probenecid is compatible with βFGF-induced regenerative responses in *in vitro* cultured human dermal fibroblasts, including migration, proliferation, and extracellular matrix-related gene expression. This approach may reveal a novel therapeutic strategy to target fibroblast dysfunction and improve repair in chronic skin wounds.

## 2. Results

### 2.1. βFGF-Induced Fibroblast Migration Is Not Modified by Panx1 Channels Inhibition

**Figure 1:**
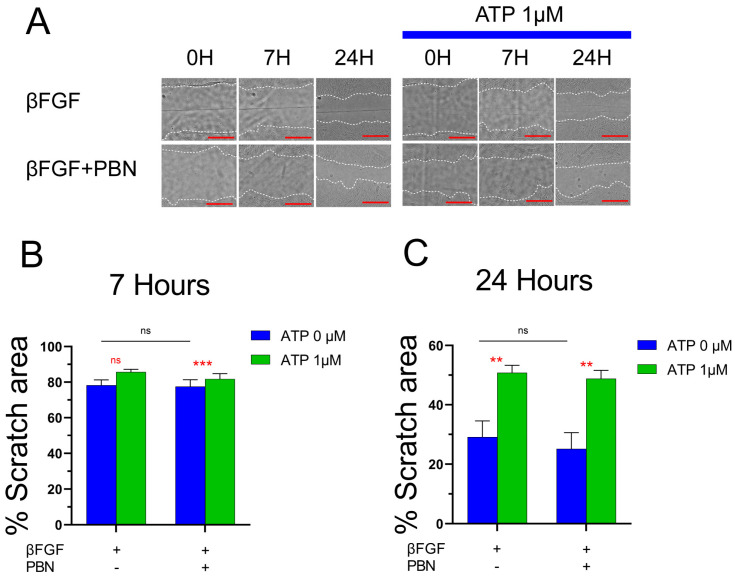
PBN did not enhance the effect of βFGF on HDFn migration. The study used an *in vitro* scratch migration assay to analyze the scratch area at 7 and 24 h. (**A**) Representative images of the scratch area at 0, 7, and 24 h in βFGF (10 ng/mL)/heparin (5 U/mL)-treated fibroblasts in the presence or absence of 1 µM eATP and/or PBN (200 μM) treatment. Scale bar: 1000 μm. Scratch area percentage in βFGF-treated fibroblasts at (**B**) 7 and (**C**) 24 h, with or without eATP and/or PBN treatment. *n* = 3, and data are presented as mean ± standard error. In all panels, “+” indicates the presence and “-” the absence of the indicated treatment condition. Statistical comparisons between different conditions were performed using ANOVA, followed by Tukey’s post hoc test. For comparisons between treatments with or without eATP (indicated by a red asterisk), Student’s *t*-test was used. Statistical significance: ** *p* < 0.01, *** *p* < 0.001; ns, not significant.

To determine whether pharmacological inhibition of Panx1 channels affects βFGF-induced fibroblast migration, we performed an *in vitro* scratch assay in human neonatal dermal fibroblast (HDFn) cultures treated with βFGF and/or PBN and evaluated wound closure at 7 and 24 h (Figure 1A and Figure S1A). In the case of βFGF (10 ng/mL) treatment, cells were supplemented with heparin (5 U/mL) to stabilize βFGF signaling, as previously described [36].

Treatment with PBN alone significantly reduced the scratch area at both time points compared with untreated control cells and heparin-treated cells (Figure S1B–F), indicating enhanced migratory activity. In contrast, treatment with βFGF did not significantly affect migration at 7 h but significantly reduced the wound area after 24 h compared with untreated control cells (Figure S1B–F). Notably, combined treatment with βFGF and PBN (200 μM) did not further enhance wound closure compared with βFGF treatment alone at either time point (Figure 1B,C).

PBN is a pharmacological inhibitor of Panx1 channels, a transmembrane channel involved in ATP release from cells into the extracellular space. Because extracellular ATP (eATP) signaling has been implicated in regulating fibroblast motility, we next evaluated the effect of exogenous ATP supplementation on migration (Figure 1A and Figure S1A). The concentration of 1 μM ATP was selected because it represented the lowest concentration at which measurable changes in fibroblast responses had previously been observed [21]. The addition of 1 µM ATP did not significantly alter wound closure in untreated control cells or in heparin-treated cultures at 7 and 24 h (Figure S1B,D). However, in PBN-treated cells, ATP supplementation prevented the reduction in scratch area induced by PBN alone, resulting in a significant difference between the two conditions (Figure S1B,D). A similar effect was observed in βFGF-treated cells at 24 h, as well as in the βFGF + PBN condition at both 7 and 24 h (Figure 1B,C).

Overall, these findings indicate that Panx1 channels inhibition does not interfere with βFGF-mediated fibroblast migration and vice versa. Instead, extracellular ATP appears to act as a negative regulator of HDFn migration under these experimental conditions.

### 2.2. Cell Proliferation Induced by βFGF and/or ATP Was Not Affected by PBN

**Figure 2:**
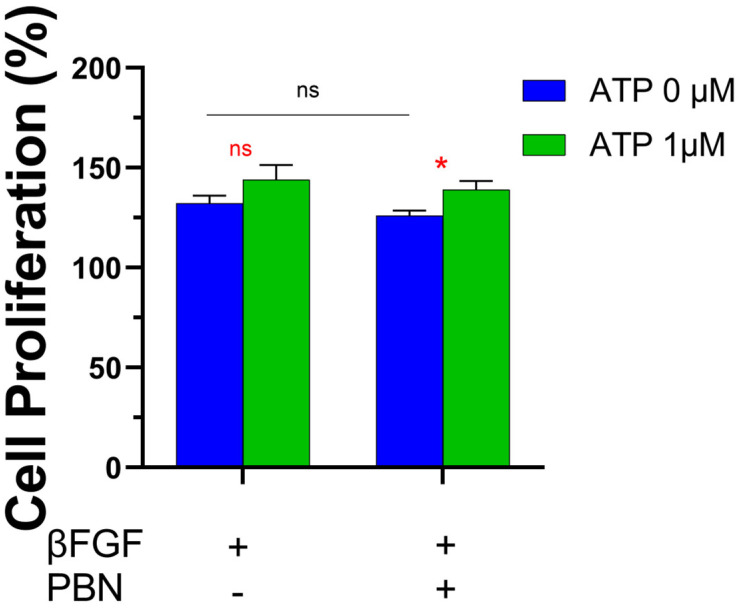
PBN did not affect proliferation in βFGF-treated HDFn cells. Cell proliferation was assessed at 72 h using the CyQUANT assay. Proliferation percentage in βFGF (10 ng/mL)/heparin (5 U/mL)-treated fibroblasts in the presence or absence of 1 µM eATP and/or PBN (200 μM) treatment. In all panels, “+” indicates the presence and “-” the absence of the indicated treatment condition. *n* = 6. Data are presented as mean ± standard error. Statistical comparisons between different conditions were performed using ANOVA, followed by Tukey’s post hoc test. For comparisons between treatments with or without 1 µM eATP (indicated by a red asterisk), Student’s *t*-test was used. Statistical significance: * *p* < 0.05; ns, not significant.

Since βFGF is a well-established regulator of fibroblast proliferation, we next investigated whether Panx1 channels inhibition influences βFGF-driven proliferative responses in HDFn cells. As expected, treatment with βFGF significantly increased cell proliferation after 72 h compared with untreated control cells (Figure S2A). In contrast, heparin alone did not significantly affect proliferation (Figure S2B). Likewise, treatment with PBN alone did not substantially alter proliferation relative to untreated control cells (Figure S2A). Importantly, combined treatment with βFGF and PBN did not differ significantly from βFGF alone (Figure 2), indicating that inhibition of Panx1 channels does not impair βFGF-induced proliferation.

Because both βFGF and PBN reduced extracellular ATP levels in HDFn cultures, we further evaluated whether exogenous ATP supplementation could modulate proliferative responses in the context of Panx1 channels inhibition. Since Panx1 channels contribute to ATP release, cells were treated with 1 μM ATP to assess whether restoring eATP signaling could influence fibroblast proliferation.

In untreated control cells, addition of 1 μM ATP significantly increased proliferation, and a similar effect was observed in PBN-treated cells (Figure S2B). Although ATP slightly increased proliferation in heparin-treated cultures, this effect did not reach statistical significance (Figure S2B). Likewise, ATP supplementation produced only a modest, non-significant increase in proliferation in βFGF-treated cells (Figure 2). Notably, PBN did not modify βFGF-induced proliferation either in the presence or absence of extracellular ATP (Figure 2).

Taken together, these findings demonstrate that pharmacological inhibition of Panx1 channels does not compromise βFGF-induced proliferative responses in HDFn cells, while eATP exerts only a modest stimulatory effect on fibroblast proliferation.

### 2.3. βFGF Modulates Extracellular Matrix-Related Gene Expression Independently of Panx1 Channels Blockade and Extracellular ATP

**Figure 3:**
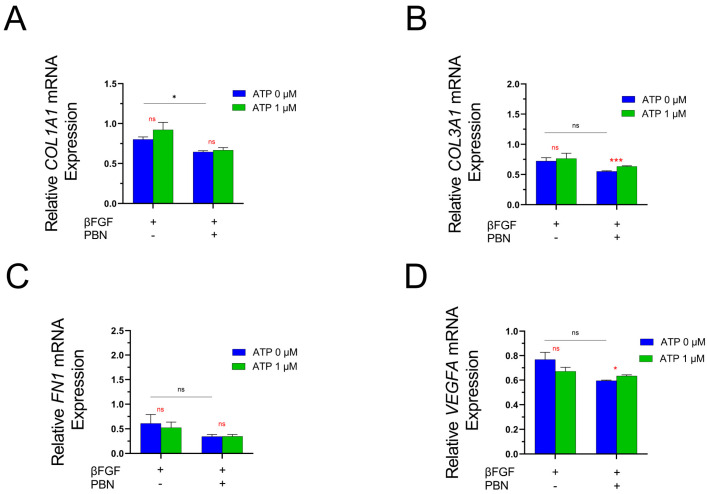
PBN does not significantly modify βFGF-induced extracellular matrix-related gene expression in HDFn cells. qRT-PCR was used to analyze gene expression after 72 h of treatment in βFGF (10 ng/mL)/heparin (5 U/mL)-treated fibroblasts in the presence or absence of 1 µM eATP and/or PBN (200 μM) treatment. (**A**) *COL1A1* mRNA expression levels. (**B**) *COL3A1* mRNA expression levels. (**C**) *FN1* mRNA expression levels. (**D**) *VEGFA* mRNA expression levels. Additionally, fibroblasts in all conditions were treated with βFGF. Treatments were evaluated in the presence or absence of 1 µM eATP and/or PBN treatment. *n* = 3. In all panels, “+” indicates the presence and “-” the absence of the indicated treatment condition. Data are presented as mean ± standard error. Statistical comparisons between different conditions were performed using ANOVA, followed by Tukey’s post hoc test. For comparisons between treatments with or without eATP (indicated by a red asterisk), Student’s *t*-test was used. Statistical significance: * *p* < 0.05, *** *p* < 0.001; ns, not significant.

Previous studies indicate that βFGF regulates extracellular matrix (ECM)-related gene expression in dermal fibroblasts, including genes associated with *COL* and *FN1* [37,38,39]. Given the central role of fibroblasts in extracellular matrix (ECM) remodeling during wound repair, we next investigated whether inhibition of Panx1 channels or eATP signaling affects βFGF-mediated regulation of ECM-related genes. To address this question, HDFn cells were treated with βFGF in the presence or absence of PBN, and transcript levels of *COL1A1*, *COL3A1*, *FN1*, and *VEGFA* were evaluated by qRT-PCR (Figure 3 and Figure S3).

Consistent with the known regulation of ECM-related genes by βFGF, treatment with βFGF significantly reduced the expression of *COL1A1*, *COL3A1*, *FN1*, and *VEGFA* compared with untreated control cells (Figure S3A–D). These findings support the notion that βFGF modulates the ECM-associated transcriptional program in HDFn cells.

Under basal conditions, PBN significantly increased *COL3A1* and *FN1* expression (Figure S3F,G), suggesting that Panx1 channels blockade alone may favor matrix-associated gene expression. Importantly, when combined with βFGF, PBN did not reverse the βFGF-induced reduction in *COL3A1*, *FN1*, and *VEGFA*-related transcripts (Figure 3B–D). The only significant interaction observed was a further decrease in *COL1A1* expression in the βFGF + PBN condition relative to βFGF treatment alone (Figure 3A).

We next evaluated the contribution of eATP to this transcriptional response. In untreated control cells, the addition of 1 μM ATP generally reduced gene expression, with a statistically significant reduction observed for *FN1* transcripts (Figure S3G). In heparin-treated cultures, ATP significantly affected *COL1A1* and *VEGFA* expression, whereas *COL3A1* and *FN1* remained unchanged (Figure S3E–H). Importantly, ATP supplementation did not reverse the βFGF-induced downregulation of *ECM*- and *VEGFA*-related genes (Figure 3A–D).

Together, these findings indicate that βFGF-mediated regulation of ECM-related genes occurs largely independently of Panx1 channels inhibition and extracellular ATP signaling.

### 2.4. βFGF Does Not Induce Transcriptional, Protein, or Membrane Redistribution Changes in Panx1 in Human Neonatal Dermal Fibroblasts

To further explore whether βFGF modulates Panx1 signaling in HDFn cells, we analyzed *PANX1* transcript levels, protein expression, and subcellular localization following βFGF treatment.

**Figure 4:**
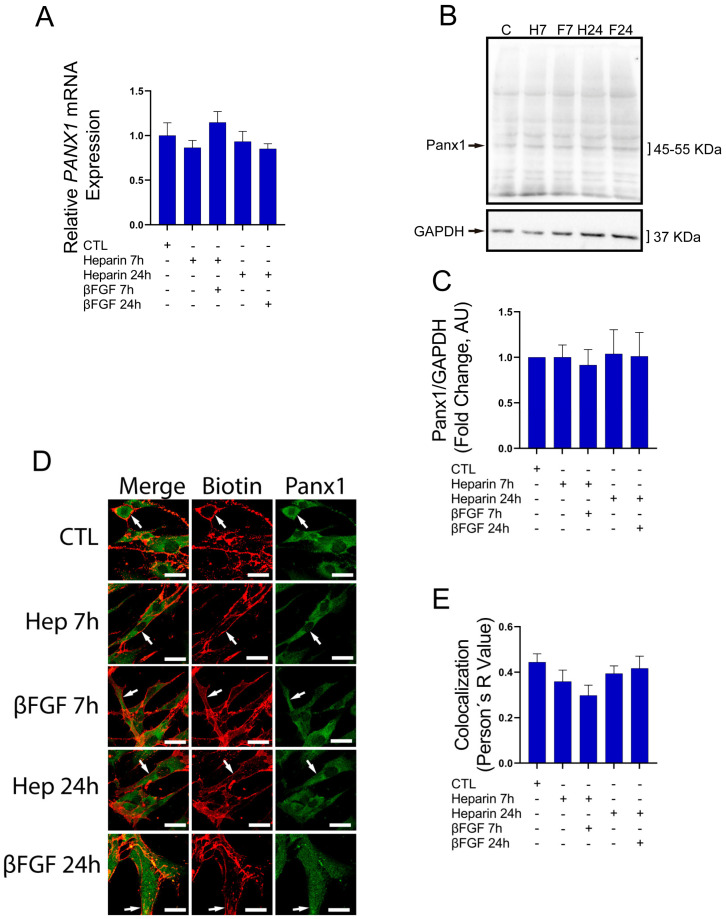
βFGF treatment does not modify Panx1 expression or localization. HDFn were treated with βFGF (10 ng/mL) and heparin (5 U/mL) for 7 h or 24 h. (**A**) Relative expression of *PANX1* determined by qPCR in untreated control cells (CTL) and cells treated with heparin or βFGF for 7 h or 24 h. *n* = 3. (**B**) Representative Western Blot of Panx1 (45–55 kDa) across experimental groups. GAPDH (37 kDa) was used as a loading control. (**C**) Quantification of the Panx1/GAPDH ratio expressed as fold change relative to control. *n* = 6. (**D**) Immunofluorescence images showing Panx1 signal (green) and plasma membrane labeling via biotinylation (red). Arrows indicate representative membrane regions. Scale bar: 30 μm. (**E**) Colocalization analysis between Panx1 and biotin using Pearson’s coefficient. CTL indicates untreated control cells. In all panels, “+” indicates the presence and “-” the absence of the indicated treatment condition. No significant changes in Panx1 expression or localization were observed under any treatment conditions. *n* = 3 different cell cultures. Data are presented as mean ± standard error. Statistical comparisons between different conditions were performed using ANOVA, followed by Tukey’s post hoc test.

qRT-PCR analysis revealed that exposure to βFGF for 7 or 24 h did not significantly alter *PANX1* mRNA expression compared with untreated or heparin-treated control cells (Figure 4A). Consistent with these findings, Western Blot analysis showed no detectable differences in Panx1 protein levels among the experimental groups at either time point (Figure 4B,C).

To determine whether βFGF influences Panx1 membrane localization, we next performed immunofluorescence analysis using anti-Panx1 antibodies and plasma membrane biotinylation. Under all experimental conditions, Panx1 exhibited a similar intracellular distribution pattern, with no apparent increase in membrane-associated signal following βFGF treatment (Figure 4D). Quantitative colocalization analysis using Pearson’s coefficient further confirmed the absence of significant differences among groups (Figure 4E).

Collectively, these results demonstrate that βFGF does not modify *PANX1* transcription, protein abundance, or plasma membrane localization in HDFn cells, suggesting that βFGF signaling does not directly regulate Panx1 expression or trafficking under the conditions evaluated.

### 2.5. βFGF Does Not Alter Panx1 Channel-Dependent ATP Release or Ca^2+^ Signaling in Human Neonatal Dermal Fibroblasts

**Figure 5:**
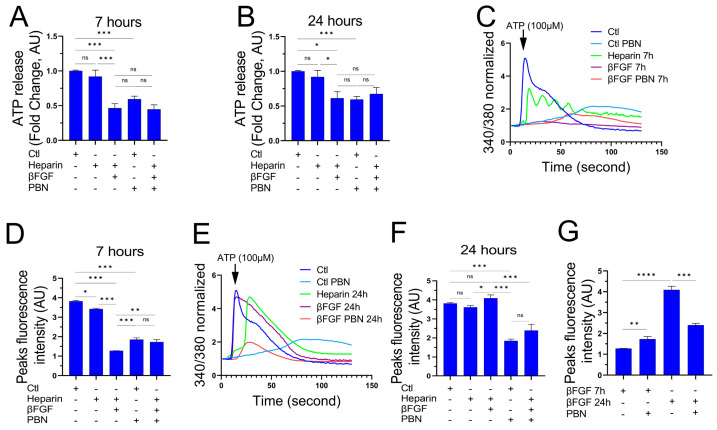
βFGF reduces ATP release and transiently modulates ATP-induced Ca^2+^ signaling in HDFn cells. Extracellular ATP levels and intracellular Ca^2+^ signaling were evaluated in HDFn treated with βFGF (10 ng/mL), heparin (5 U/mL), PBN (200 μM), or the indicated combinations for 7 or 24 h. (**A**,**B**) Extracellular ATP release was quantified after (**A**) 7 h and (**B**) 24 h of treatment and expressed as fold change relative to the control condition. *n* = 6 independent cultures. (**C**–**G**) Intracellular Ca^2+^ signaling was assessed by quantifying the amplitude of fluorescence peaks following stimulation with ATP (100 μM). (**C**) Representative ATP-induced Ca^2+^ peak amplitudes under the indicated experimental conditions at 7 h. (**D**) Quantification of ATP-induced Ca^2+^ peak amplitudes in cells treated with βFGF for 7 h. (**E**) Representative ATP-induced Ca^2+^ peak amplitudes under the indicated experimental conditions at 24 h. (**F**) Quantification of ATP-induced Ca^2+^ peak amplitudes in cells treated with βFGF for 24 h. (**G**) Direct comparison of Ca^2+^ peak amplitudes between βFGF-treated cells at 7 and 24 h. For Ca^2+^ imaging experiments. *n* = 3 independent cell cultures, with 10 cells analyzed per culture. CTL indicates untreated control cells. In all panels, “+” indicates the presence and “-” the absence of the indicated treatment condition. Data are presented as mean ± standard error. Statistical comparisons were performed using one-way ANOVA followed by Tukey’s post hoc test. Statistical significance is indicated as * *p* < 0.05, ** *p* < 0.01, *** *p* < 0.001, **** *p* < 0.0001; ns, not significant.

Because Panx1 channels are major regulators of extracellular ATP release and purinergic signaling, we finally investigated whether βFGF modulates Panx1 channels-dependent ATP release and intracellular Ca^2+^ dynamics in HDFn cells. eATP levels and ATP-evoked intracellular Ca^2+^ responses were analyzed after 7 and 24 h of treatment with βFGF supplemented with heparin (Figure 5A–G).

βFGF treatment significantly reduced extracellular ATP levels at both 7 and 24 h compared with untreated control cells (Figure 5A,B). A similar reduction was observed in PBN-treated cultures, whereas heparin alone did not affect extracellular ATP release. Moreover, combined treatment with βFGF and PBN did not produce additional inhibition beyond that observed with either treatment alone, and no significant differences were detected among βFGF, PBN, or βFGF + PBN conditions at either time point (Figure 5A,B).

Because extracellular ATP is a key mediator of purinergic Ca^2+^ signaling, we next evaluated ATP-induced intracellular Ca^2+^ responses (Figure 5C,E). At 7 h, βFGF significantly reduced the amplitude of ATP-evoked Ca^2+^ transients compared with control cells (Figure 5D). However, after 24 h, Ca^2+^ responses recovered to levels comparable to controls, although they remained significantly higher than those observed in heparin-treated cells (Figure 5F). PBN treatment reduced Ca^2+^ peak amplitudes at both time points. Interestingly, at 7 h, Ca^2+^ responses in PBN-treated cells were slightly but significantly higher than those observed in βFGF-treated cells, whereas the opposite tendency was detected after 24 h (Figure 5D,F). No significant differences were observed between PBN and βFGF + PBN conditions (Figure 5D,F).

Direct comparison between βFGF-treated groups confirmed a temporal modulation of intracellular Ca^2+^ signaling, with significantly different responses at 7 and 24 h (Figure 5G).

Overall, these findings indicate that βFGF reduces extracellular ATP release and transiently modulates ATP-dependent Ca^2+^ signaling in HDFn cells. However, because βFGF did not alter *PANX1* expression or membrane localization, these effects are unlikely to result from direct regulation of Panx1 channels activity and may instead involve downstream modulation of purinergic signaling pathways.

## 3. Discussion

A comprehensive understanding of the role of Panx1 channels function in wound healing is crucial for developing novel therapeutic strategies for chronic wounds, in which inflammation, proliferation, and tissue remodeling are dysregulated. In the present study, we found that treatment with PBN, a pharmacological inhibitor of Panx1 channels, did not significantly alter βFGF activity in HDFn cells, except for *COL1A1* transcription. This key growth factor governs fibroblast migration, proliferation, and differentiation during cutaneous repair. These findings suggest that PBN-mediated inhibition of Panx1 channels does not directly interfere with the βFGF signaling pathway.

Regarding cell migration, our results indicate that βFGF at 10 ng/mL increases fibroblast migration. This finding is consistent with multiple studies demonstrating that βFGF enhances fibroblast migration under various conditions, including pathological states such as hyperglycemia, which restores migration by reactivating the JNK pathway [40]. Moreover, βFGF-controlled release systems have demonstrated improved fibroblast migration and accelerated tissue regeneration [41], mediated by pathways such as PI3K/Akt, Rac1, and JNK, as well as cooperative routes including Hedgehog and NF-κB [42].

In parallel, Panx1 channels inhibition significantly increased fibroblast migration, consistent with studies reporting that Panx1 silencing or pharmacological inhibition enhances dermal fibroblast motility *in vitro* [21] and in Panx1-knockout mouse models [19]. Although the precise mechanism remains unclear, it is hypothesized that inhibition of Panx1 channels promotes cytoskeletal remodeling and the formation of membrane protrusions [21]. Furthermore, extracellular ATP partially reversed the promigratory effects induced by Panx1 channels inhibition, suggesting that eATP may negatively regulate fibroblast migration under these experimental conditions. The combined treatment with βFGF and PBN did not result in significant alterations in the migratory response, suggesting that these pathways act independently.

We also demonstrated that βFGF treatment significantly enhances HDFn proliferation. Studies have previously reported this effect, linking βFGF action to the activation of intracellular signaling pathways such as ERK1/2 and JNK, which are crucial for cell cycle progression and survival [43]. In contrast, no significant increase in proliferation was observed in fibroblasts treated solely with PBN. Current evidence suggests that Panx1 channels may suppress the cell cycle in dermal fibroblasts, likely via ATP release and activation of purinergic receptors. This effect, however, appears to be highly cell-type-dependent, as Panx1 deficiency does not alter keratinocyte proliferation [19]. In contrast, tumor cells such as melanocytes can promote proliferation via the Wnt/β-catenin pathway [44]. Our results further demonstrate that combined treatment with βFGF and Panx1 channels inhibition did not enhance the proliferative response, suggesting no synergistic interaction between the two pathways.

Additionally, we found that extracellular ATP at 1 μM promotes fibroblast proliferation, consistent with reports that moderate ATP concentrations (1–100 μM) activate P2 purinergic receptors and stimulate signaling pathways, such as MAPK/ERK and PI3K/Akt [45]. In contrast, high ATP concentrations (>1 mM) have been associated with cytotoxic and differentiation-inducing effects [46], supporting the concept of ATP as a biphasic extracellular signal.

Concerning gene expression, we found that βFGF downregulates *VEGFA* transcript levels in human dermal fibroblasts. This observation contrasts with previous studies conducted in murine embryonic fibroblast lines, which reported upregulation of VEGF expression [47]. The discrepancy may be attributed to cell origin and physiological context. Indeed, βFGF has been shown to reduce VEGF expression in specific pathological models, such as preeclampsia-induced placentas in rats [48], and its angiogenic effects are predominantly mediated via endothelial cells [33]. In addition, excessive *VEGFA* expression and angiogenesis have been associated with pathological fibrotic skin remodeling, including hypertrophic scarring, where increased *VEGFA* levels, microvascular density, and enhanced VEGF immunoreactivity have been reported in proliferative scar tissue [49,50,51]. Moreover, our findings indicate that βFGF decreases the expression of *COL1A1*, *COL3A1*, and *FN1* in human dermal fibroblasts, consistent with previous transcriptomic and gene expression studies reporting modulation of extracellular matrix-associated genes by βFGF [37,38,39]. These findings suggest that βFGF may contribute to extracellular matrix remodeling under the experimental conditions evaluated.

We observed that PBN treatment altered gene expression, resulting in significant increases in *COL3A1* and *FN1* levels. In contrast, no significant differences were observed in *VEGFA* and *COL1A1* expression. Moreover, PBN downregulated *COL1A1* expression in HDFs treated with βFGF, which may be associated with altered purinergic signaling and downstream calcium-dependent pathways that regulate the ECM. This mechanism is consistent with previous findings showing that ATP release through hemichannels in fibroblasts modulates calcium-dependent signaling pathways involved in ECM remodeling [52]. Given that calcium elevation has been associated with increased ECM genes [53,54], downregulation of *COL1A1* may reflect a negative regulatory effect of Panx1 channels inhibition on calcium-mediated profibrotic signaling.

Finally, we found that ATP at 1 μM reduces the expression of *VEGFA*, *COL1A1*, *COL3A1*, and *FN1*. Although eATP has been reported to promote matrix production at higher concentrations (10–100 μM) [55]. Our data suggest that at low concentrations, it may exert an inhibitory effect, possibly due to selective activation of inhibitory purinergic receptors or insufficient activation of the PI3K/Akt signaling pathway.

Previous evidence suggests that various growth factors can influence Panx1 channels activity. Specifically, FGF-1 has been shown to promote the opening of Panx1 channels in spinal astrocytes through an autocrine/paracrine mechanism that depends on ATP release and the activation of P2×7 receptors, thereby increasing cellular permeability [56]. This effect has been observed in both cultured cells and acute spinal cord slices, in which activation of the Panx1 channels by FGF-1 has been associated with neuroinflammatory responses. Similarly, treating endothelial cells with TNF-α has been reported to enhance Panx1 channels function and promote extracellular ATP release, reinforcing its role in vascular inflammation [57]. Although our results demonstrate that βFGF treatment reduces eATP levels, we cannot conclusively determine whether this effect is mediated by inhibition of Panx1 channels activity. To date, no studies have directly shown that βFGF functionally modulates Panx1 channels. Conversely, a study in tanycytes has demonstrated that βFGF promotes ATP release through connexin-43 hemichannels and enhances extracellular purinergic signaling [58]. In this context, the decrease in eATP observed in human dermal fibroblasts suggests that the effects of βFGF may depend on cell–type–specific mechanisms that differ from those in central nervous system models. Overall, these findings indicate that while βFGF can influence eATP levels, the role of Panx1 channels in this process remains uncertain. It is important to acknowledge that the experimental time points evaluated in the present study may not capture rapid or transient Panx1 channels-dependent signaling events, since ATP release and intracellular Ca^2+^ responses can occur within seconds or minutes following stimulation. Therefore, transient early signaling events induced by βFGF cannot be excluded under the present experimental conditions.

Our results demonstrate that βFGF treatment temporally modulates intracellular Ca^2+^ signaling, as evidenced by a reduction in Ca^2+^ signal amplitude at 7 h, followed by a return to levels comparable to those in control conditions at 24 h. This pattern is consistent with previous studies showing that βFGF induces rapid, transient increases in intracellular Ca^2+^ by activating PLCγ1 and promoting InsP_3_ production, thereby promoting Ca^2+^ release from the endoplasmic reticulum. In human endothelial cells (HUVECs), βFGF at 100 ng/mL elicits a Ca^2+^ peak within approximately 1 min, followed by rapid signal attenuation [59]. Similarly, in embryonic rat neural stem cells, βFGF treatment (10 ng/mL) induces a rapid increase in intracellular Ca^2+^ associated with proliferative responses [60]. In addition, studies in satellite cells and MDCK-F cells have reported βFGF-induced Ca^2+^ elevations that depend primarily on TRPC-type channels [61,62]. Collectively, these findings support the notion that βFGF-induced Ca^2+^ signaling occurs predominantly during the early phases of stimulation, and that subsequent signal attenuation may account for the decrease observed at 7 h and the recovery at 24 h in our experimental model. Accordingly, one possible explanation, based on the evidence described above, is that the effects of βFGF on intracellular Ca^2+^ dynamics in HDFn cells are mediated primarily through FGFR-dependent intracellular pathways rather than by direct regulation of Panx1 channels expression or trafficking. Future studies should experimentally evaluate these pathways to further clarify the mechanisms underlying βFGF-mediated purinergic responses in dermal fibroblasts.

On the other hand, although ATP released into the extracellular space via Panx1 channels has been shown to activate purinergic receptors and promote Ca^2+^ entry [63], there is currently no direct evidence linking βFGF to the functional activation of Panx1 channels. While FGF1 has been reported to increase intracellular Ca^2+^ through Panx1 channels activation and associated purinergic signaling [64], our study did not reveal evidence of a significant contribution of Panx1 channels to βFGF-induced Ca^2+^ modulation. These findings suggest that the observed effects are primarily mediated by intracellular mechanisms downstream of FGFR activation rather than by ATP release through Panx1 channels.

A major limitation of the present study is the exclusive use of *in vitro* fibroblast cultures, which do not fully recapitulate the complexity of chronic wound microenvironments, including inflammatory cell interactions, vascular components, extracellular matrix remodeling, hypoxia, and proteolytic activity, all of which critically influence the progression of wound healing [65]. Although probenecid is widely used as a pharmacological inhibitor of Panx1 channels, it is important to acknowledge that this compound may exert additional effects beyond selective Panx1 channels blockade. Previous studies have reported that probenecid modulates P2X7 receptor-associated responses at concentrations near 1 mM [66]. However, the concentration used in the present study (200 µM) is substantially lower and has previously been shown to effectively inhibit Panx1 channels-associated ATP release and dye uptake in human dermal fibroblasts while enhancing fibroblast migration [21]. Importantly, similar promigratory effects were observed using complementary approaches, including the mimetic peptide 10Panx and *PANX1* siRNA-mediated silencing [21], supporting the interpretation that the observed responses are primarily attributable to Panx1 channels inhibition under these experimental conditions. In addition, because the present study relied exclusively on pharmacological inhibition using probenecid, which is known to exert off-target effects beyond Panx1 channels, the observed responses cannot be definitively attributed to selective Panx1 channels inhibition. Therefore, although our findings provide preliminary evidence regarding the compatibility between probenecid-mediated Panx1 channels inhibition and βFGF-induced fibroblast responses, future studies using complementary genetic approaches and *in vivo* chronic wound models will be necessary to further establish the mechanistic and translational relevance of these observations.

## 4. Materials and Methods

### 4.1. Cell Culture

Human neonatal foreskin dermal fibroblasts (HDFn) were commercially obtained (C0045C, GIBCO, Grand Island, NY, USA). Cells were cultured in DMEM supplemented with 10% fetal bovine serum (FBS) (Cytiva, Amersham, UK), penicillin (100 U/mL), streptomycin (10 μg/mL) (Thermo Scientific Inc^®^, Waltham, MA, USA), and 2 mM GlutaMAX (Thermo Scientific Inc^®^). Cell cultures were maintained in an incubator (Memmert, Schwabach, Germany) under a humidified atmosphere at 37 °C with 5% CO_2_.

### 4.2. Inhibition of Panx1 Channels

To assess the contribution of Panx1 channels, we utilized 200 µM probenecid (PBN) (Thermo Fisher Scientific, Waltham, MA, USA), a non-selective Panx1 channels inhibitor. The concentration of probenecid (200 μM) was selected based on previous studies demonstrating effective modulation of Panx1 channels-sensitive signaling in dermal fibroblasts without significant cytotoxicity [21].

### 4.3. In Vitro Scratch Migration Assay

HDFn cells were cultured in 4-well plates until reaching confluence. A linear wound was then created using a P-1000 pipette tip, and cells were cultured in DMEM supplemented with 1% FBS, βFGF (10 ng/mL), heparin (5 U/mL), 1µM ATP, and PBN. Images were taken at 0, 7, and 24 h using an Axiovert 135M microscope (Zeiss, Germany) equipped with a 40X objective. Image acquisition was performed with an Axiocam 305 camera (Zeiss, Oberkochen, Germany) and ZEN 3.10 software (Zeiss, Germany). Image analysis was conducted using ImageJ software (version 1.49v; NIH, Bethesda, MD, USA). The concentration of βFGF (10 ng/mL) was selected based on previous studies demonstrating that this dose effectively promotes fibroblast migration, proliferation, and extracellular matrix-related responses without inducing overt cytotoxicity or excessive differentiation [67,68]. Moreover, previous studies evaluating fibroblast migration and regenerative responses have commonly used this concentration as a biologically active threshold for βFGF-mediated signaling [40,69].

### 4.4. Cell Proliferation Assay

Cell proliferation in HDFn cultures treated with βFGF (10 ng/mL), heparin (5 U/mL), 1 µM ATP, and PBN was assessed using the CyQUANT™ proliferation assay kit (Invitrogen, Waltham, MA, USA). HDFn cells were seeded at 2500 cells per well in 96-well plates and cultured for 72 h. Proliferation was then measured according to the manufacturer’s instructions. Fluorescence was measured using a Varioskan microplate reader (Thermo Scientific Inc.) with excitation at 480 nm and emission at 520 nm.

### 4.5. Quantitative Real-Time Polymerase Chain Reaction (qRT-PCR)

The expression levels of *VEGFA*, *COL1A1*, *COL3A1*, and *FN1* transcripts were evaluated by qRT-PCR after 72 h of treatment with βFGF (10 ng/mL), heparin (5 U/mL), ATP (1 µM), and/or probenecid (PBN, 200 µM). In contrast, *PANX1* transcript expression was independently evaluated after 7 or 24 h of βFGF treatment to determine whether βFGF modulates *PANX1* expression at early and intermediate time points. Total RNA was extracted using the E.Z.N.A.^®^ Total RNA Kit (Omega Bio-Tek, Norcross, GA, USA). cDNA was synthesized using the M-MLV Reverse Transcriptase Kit (Bioneer, Daejeon, Republic of Korea). mRNA expression was quantified using the SYBR Green Supermix Kit (Bio-Rad, CA, USA) on a QuantStudio 3 Real-Time PCR system (Applied Biosystems, Waltham, MA, USA). *GAPDH* was used as a housekeeping gene. The primer sequences are listed in Table 1.

### 4.6. Western Blot

Panx1 protein levels were assessed by Western Blot in HDFn cells treated with βFGF (10 ng/mL) and heparin (5 U/mL) for 7 or 24 h. HDFn cultures were first lysed and homogenized in a lysis buffer containing a protease inhibitor cocktail. Protein concentration was determined using the Qubit^®^ Protein Assay Kit (Thermo Scientific, Rockford, IL, USA). A total of 40 µg of protein was loaded onto a 10% SDS-PAGE gel, separated by electrophoresis, and transferred onto a PVDF membrane for immunoblotting. Membranes were incubated with primary antibodies against Panx1 (rabbit, 1:500; MAB7097, R&D Systems) and GAPDH (rabbit, 1:10,000; 181602, Abcam, Cambridge, UK) as a loading control. After washing, membranes were incubated with HRP-conjugated secondary antibodies (1:10,000; Jackson ImmunoResearch, West Grove, PA, USA; 1:5000) and visualized using an enhanced chemiluminescence (ECL) kit (Bio-Rad, Hercules, CA, USA). Densitometric analysis was performed using ImageJ software (version 1.49v; NIH, Bethesda, MD, USA).

### 4.7. Colocalization Analysis

HDFn cultures, either untreated or treated with βFGF (10 ng/mL) plus heparin (5 U/mL) for 7 or 24 h, were used for immunofluorescence analysis. To label the plasma membrane, cells were incubated with Ez-Link NHS-10-Biotin (0.5 mg/mL; A39257, Thermo Scientific) for 20 min and washed with glycine (1.5 mM, pH 8). Cultures were then fixed with 4% paraformaldehyde (PFA) for 30 min, permeabilized with 1% Triton X-100 in PBS, and blocked with 2% normal goat serum in PBS, as previously described [73]. Panx1 was detected using a primary antibody against Panx1 (rabbit, 1:200; 455100, Invitrogen) followed by Alexa Fluor-conjugated secondary antibodies. Avidin-Cy3 (E4142, Sigma Aldrich, MA, USA) was used for biotin detection. Fluorescence images were acquired with a Leica Stellaris 5 confocal microscope and processed using Leica Application Suite X (LAS X) software. Colocalization analysis was performed using ImageJ software (version 1.49v; NIH, Bethesda, MD, USA).

### 4.8. Extracellular ATP Assay

Extracellular ATP levels were assessed in HDFn cultures treated with βFGF. First, HDFn cells were cultured in 96-well plates with βFGF (10 ng/mL) and heparin (5 U/mL) for 7 and 24 h. The 7 h and 24 h time points were selected to evaluate sustained changes in extracellular ATP release and purinergic signaling associated with fibroblast regenerative responses rather than rapid transient signaling events occurring immediately after βFGF stimulation. Heparin was included as a stabilizing cofactor for βFGF signaling [36]. The culture medium was then removed and replaced with HBSS for 30 min, during which cells were incubated with probenecid (PBN). Extracellular ATP levels were quantified using the Luciferin-Luciferase ATP Determination Assay Kit (Thermo Fisher Scientific, Waltham, MA, USA). Luminescence was measured using a Varioskan microplate reader (Thermo Fisher Scientific, Cambridge, MA, USA).

### 4.9. Intracellular Ca^2+^ Signal Measurement

Intracellular Ca^2+^ levels in HDFn cells treated with βFGF (10 ng/mL) and heparin (5 U/mL) for 7 or 24 h were measured using a Ca^2+^-sensitive fluorescent dye. As previously described [48], HDFn cultures were loaded with 5 µM fluo-4 AM (Invitrogen) in the presence of Pluronic F-127 (Invitrogen) at a 1:1 ratio relative to the fluo-4 AM. Fluorescence images were acquired by alternating excitation at 488 nm and recording emission at 510 nm. Baseline fluorescence was recorded for 3 min, after which cells were stimulated with 100 µM ATP (Thermo Scientific Inc.), and Ca^2+^ responses were recorded for an additional 5 min. For experiments involving Panx1 channels blockade, cultures were treated with PBN throughout the recording period.

### 4.10. Statistical Analysis

Data are presented as mean ± standard error in graphical format. Each set of experiments included 3 to 9 replicates obtained from three independent cultures. Data were collected and organized using Microsoft Excel. Statistical analyses and graph generation were performed using GraphPad Prism (version 9, GraphPad Software, Inc., San Diego, CA, USA). The Shapiro–Wilk test was used to assess the normality of the data. Statistical significance was determined using one-way ANOVA followed by Tukey’s post hoc test. A *p*-value < 0.05 was considered statistically significant.

## 5. Conclusions

Our findings demonstrate that pharmacological inhibition of Panx1 channels with PBN does not compromise βFGF-driven regenerative responses in human dermal fibroblasts. Specifically, PBN did not significantly alter βFGF-induced migration, proliferation, or most extracellular matrix-related transcriptional responses, despite βFGF-mediated modulation of extracellular ATP release and intracellular Ca^2+^ signaling occurring without detectable changes in *PANX1* expression or membrane localization. These findings indicate that pharmacological inhibition of Panx1 channels by PBN does not interfere with βFGF-driven signaling pathways involved in fibroblast regenerative responses during wound repair.

Nevertheless, some limitations of the present study should be acknowledged. Because probenecid may exert off-target effects beyond Panx1 inhibition, the observed responses cannot be exclusively attributed to Panx1 channels blockade, and future studies using genetic approaches or more selective inhibitors will be necessary. In addition, although changes in extracellular matrix-related gene expression were identified, these findings were not validated at the protein or functional level. Finally, the exclusive use of *in vitro* fibroblast cultures does not fully recapitulate the complexity of chronic wound microenvironments. In addition, because chronic wound healing involves complex interactions among fibroblasts, inflammatory cells, endothelial cells, extracellular matrix remodeling, hypoxia, and proteolytic signaling, the simplified monolayer fibroblast model used in this study cannot fully reproduce the multicellular microenvironment present *in vivo*. Therefore, additional *in vivo* studies will be required to further establish the mechanistic and translational relevance of combining Panx1 channels inhibition with βFGF-driven regenerative approaches in chronic wound healing.

## Figures and Tables

**Table 1:** Primer list.

	Primer	
Gen	Sequence	Reference
*PANX1*	Forward: CTGTGGACAAGATGGTCACG Reverse: CAGCAGGATGTAGGGGAAAA	[70]
*VEGFA*	Forward: TGCAGATTATGCGGATCAAACC Reverse: TGCATTCACATTTGTTGTGCTGTAG	[71]
*COL1A1*	Forward: CTGTAAACTCCCTCCATCCC Reverse: GTCCATGTGAAATTGTCTCCC	[72]
*COL3A1*	Forward: CTGGGGAATGGAGCAAAAC Reverse: AAAGCAAACAGGGCCAAC	[72]
*FN1*	Forward: GGACCAGGACCAACAAAAAC Reverse: AGACACTAACCACATACTCCAC	[72]
*GAPDH*	Forward: ATGGGGAAGGTGAAGGTCG Reverse: GAGGTCAATGAAGGGGTCAT	[70]

## Data Availability

The original contributions presented in this study are included in the article/Supplementary Material. Further inquiries can be directed to the corresponding author.

## Supplementary Materials

The following supporting information can be downloaded at: https://www.mdpi.com/article/10.3390/ijms27125155/s1.

## Abbreviations

AKT	Protein kinase B
ATP	Adenosine Triphosphate
COL I	Collagen Type I
COL III	Collagen type III
DMEM	Dulbecco’s Modified Eagle Medium
ECL	Enhanced Chemiluminescence
ECM	Extracellular Matrix
ERK	Extracellular Signal-Regulated Kinase
FBS	Fetal Bovine Serum
βFGF	Basic Fibroblast Growth Factor
FGF1	Fibroblast Growth Factor 1
FGFR	Fibroblast Growth Factor Receptor
FN	Fibronectin
GAPDH	Glyceraldehyde-3-Phosphate Dehydrogenase
HBSS	Hank’s Balanced Salt Solution
HDF	Human Dermal Fibroblasts
HRP	Horseradish Peroxidase
JNK	c-Jun N-terminal Kinase
MAPK	Mitogen-Activated Protein Kinase
MDCK	Madin–Darby Canine Kidney
NF	Nuclear Factor
NHS	N-Hydroxysuccinimide
PAGE	Polyacrylamide Gel Electrophoresis
PBN	Probenecid
PBS	Phosphate-Buffered Saline
PCR	Polymerase Chain Reaction
PFA	Paraformaldehyde
PVDF	Polyvinylidene Fluoride
qRT-PCR	Quantitative Reverse Transcription Polymerase Chain Reaction
RAF	Rapidly Accelerated Fibrosarcoma Kinase
RAS	Rat Sarcoma GTPase
RNA	Ribonucleic Acid
SDS	Sodium Dodecyl Sulfate
SYBR	SYBR Green Fluorescent Dye
TNF	Tumor Necrosis Factor
TRPC	Transient Receptor Potential Canonical Channel
VEGF	Vascular Endothelial Growth Factor
