# Supplementary material for: Probenecid-Mediated Pannexin-1 Inhibition Preserves βFGF-Driven Regenerative Responses in Human Dermal Fibroblasts
Source: Int J Mol Sci. 2026 Jun 6;27(12):5155. doi: 10.3390/ijms27125155 (PMC13299604; doi:10.3390/ijms27125155)
Supplement: Supplementary file 1 [file ijms-27-05155-s001.zip › ijms-4283907-Suplementary Figures REVISED 2.pdf]

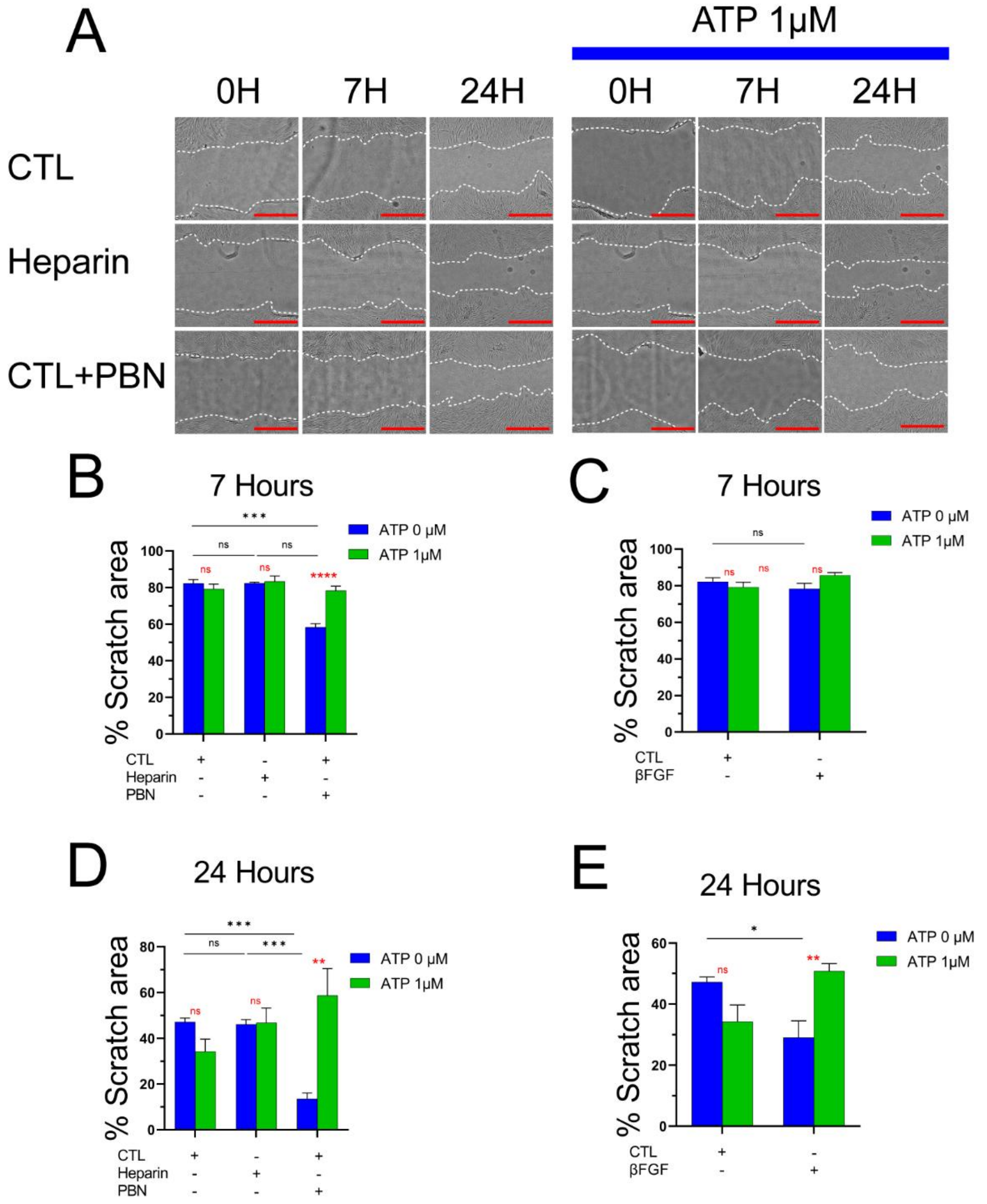

Supplementary Figure S1. Additional analyses of human neonatal dermal fibroblast migration under untreated control, heparin, βFGF, and PBN-treated conditions. The study used an *in vitro* scratch migration assay to analyze the scratch area at 7 and 24 h. **(A)** Representative images of the scratch area at 0, 7, and 24 h in untreated control fibroblasts, heparin-treated fibroblasts, βFGF (10 ng/mL)/heparin (5 U/mL)-treated fibroblasts, and PBN (200 μM)-treated fibroblasts, in the presence or absence of 1 μM eATP. Scale bar: 1000 μm. Scratch area percentage at 7 h in **(B)** untreated control and heparin-treated fibroblasts and **(C)** βFGF-treated fibroblasts with or without PBN treatment. Scratch area percentage at 24 h in **(D)** untreated control and heparin-treated fibroblasts and **(E)** βFGF-treated fibroblasts with or without PBN treatment. Treatments were evaluated in the presence or absence of 1 μM eATP. $n = 3$, and data are presented as mean ± standard error. Ctl indicates untreated control cells. In all panels, "+" indicates the presence and "-" the absence of the indicated treatment condition. Statistical comparisons between different conditions were performed using ANOVA, followed by Tukey's post

hoc test. For comparisons between treatments with or without eATP (indicated by a red asterisk), the Student's t-test was used. Statistical significance: *p < 0.05, **p < 0.01, ***p < 0.001; ns, not significant.

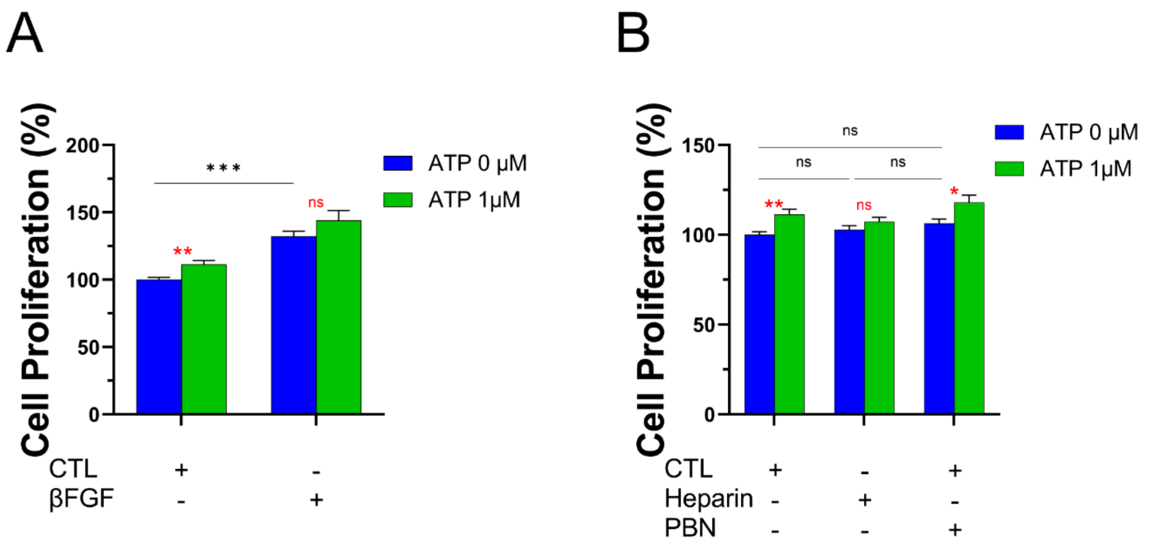

Supplementary Figure S2. Additional analyses of human neonatal dermal fibroblast proliferation under untreated control, heparin, βFGF, and PBN-treated conditions. Cell proliferation was assessed at 72 h using the CyQUANT assay. Proliferation percentage in **(A)** βFGF (10 ng/mL)/heparin (5 U/mL)-treated fibroblasts with or without PBN (200 μM) treatment and **(B)** untreated control and heparin-treated fibroblasts. Treatments were evaluated in the presence or absence of 1 μM eATP. $n = 6$. Data are presented as mean ± standard error. Ctl indicates untreated control cells. In all panels, "+" indicates the presence and "-" the absence of the indicated treatment condition. Statistical comparisons between different conditions were performed using ANOVA, followed by Tukey's post hoc test. For comparisons between treatments with or without eATP (indicated by a red asterisk), Student's t-test was used. Statistical significance: *p < 0.05, **p < 0.01, ***p < 0.001; ns, not significant.

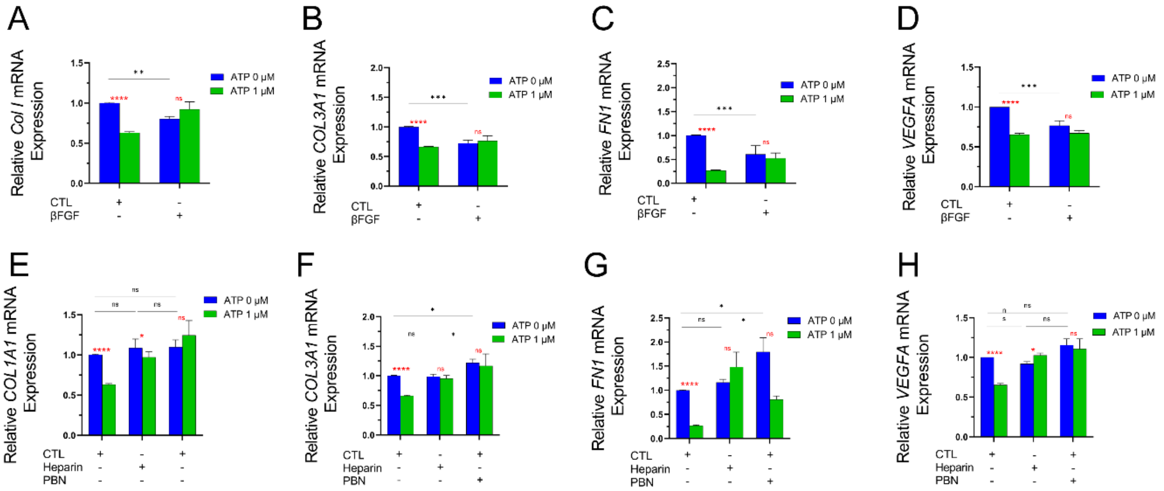

Supplementary Figure S3. Additional analyses of extracellular matrix-related gene expression in untreated control, heparin, βFGF, and PBN-treated human neonatal dermal fibroblasts. qRT-PCR was used to analyze gene expression 72 h post-treatment. **(A)** *COL1A1*, **(B)** *COL3A1*, **(C)** *FN1*, and **(D)** VEGFA mRNA expression levels in untreated control and heparin-treated fibroblasts. **(E)** *COL1A1*, **(F)** *COL3A1*, **(G)** *FN1*, and **(H)** *VEGFA* mRNA expression levels in βFGF (10 ng/mL)/heparin (5 U/mL)-treated fibroblasts with or without PBN (200 µM) treatment. Treatments were evaluated in the presence or absence of 1 µM eATP. $n = 3$. Data are presented as mean ± standard error. Ctl indicates untreated control cells. In all panels, "+" indicates the presence and "-" the absence of the indicated treatment condition. Statistical comparisons between different conditions were performed using ANOVA, followed by Tukey's post hoc test. For comparisons between treatments with or without eATP (indicated by a red asterisk), the Student's t-test was used. Statistical significance: *p < 0.05, **p < 0.01, ***p < 0.001, ****p < 0.0001; ns, not significant.
